# Ethylene induces combinatorial effects of histone H3 acetylation in gene expression in *Arabidopsis*

**DOI:** 10.1186/s12864-017-3929-6

**Published:** 2017-07-17

**Authors:** Likai Wang, Fan Zhang, Siddharth Rode, Kevin K. Chin, Eun Esther Ko, Jonghwan Kim, Vishwanath R. Iyer, Hong Qiao

**Affiliations:** 1 Institute for Cellular and Molecular Biology, The University of Texas at Austin, Austin, 78712 Texas USA; 2 The Center for Systems and Synthetic Biology, The University of Texas at Austin, Austin, 78712 Texas USA; 3 Department of Molecular Biosciences, The University of Texas at Austin, Austin, Texas 78712 USA

**Keywords:** Histone acetylation, Combinatorial effects, Ethylene, *Arabidopsis*

## Abstract

**Background:**

Histone acetylation and deacetylation are essential for gene regulation and have been implicated in the regulation of plant hormone responses. Many studies have indicated the role of histone acetylation in ethylene signaling; however, few studies have investigated how ethylene signaling regulates the genomic landscape of chromatin states. Recently, we found that ethylene can specifically elevate histone H3K14 acetylation and the non-canonical histone H3K23 acetylation in etiolated seedlings and the gene activation is positively associated with the elevation of H3K14Ac and H3K23Ac in response to ethylene. To assess the role of H3K9, H3K14, and H3K23 histone modifications in the ethylene response, we examined how ethylene regulates histone acetylation and the transcriptome at global level and in ethylene regulated genes both in wild type (Col-0) and *ein2-5* seedlings.

**Results:**

Our results revealed that H3K9Ac, H3K14Ac, and H3K23Ac are preferentially enriched around the transcription start sites and are positively correlated with gene expression levels in Col-0 and *ein2-5* seedlings both with and without ethylene treatment. In the absence of ethylene, no combinatorial effect of H3K9Ac, H3K14Ac, and H3K23Ac on gene expression was detected. In the presence of ethylene, however, combined enrichment of the three histone acetylation marks was associated with high gene expression levels, and this ethylene-induced change was EIN2 dependent. In addition, we found that ethylene-regulated genes are expressed at medium or high levels, and a group of ethylene regulated genes are marked by either one of H3K9Ac, H3K14Ac or H3K23Ac. In this group of genes, the levels of H3K9Ac were altered by ethylene, but in the absence of ethylene the levels of H3K9Ac and peak breadths are distinguished in up- and down- regulated genes. In the presence of ethylene, the changes in the peak breadths and levels of H3K14Ac and H3K23Ac are required for the alteration of gene expressions.

**Conclusions:**

Our study reveals that the plant hormone ethylene induces combinatorial effects of H3K9Ac, K14Ac and K23Ac histone acetylation in gene expression genome widely. Further, for a group of ethylene regulated genes, in the absence of ethylene the levels and the covered breadths of H3K9Ac are the preexist markers for distinguishing up- and down- regulated genes, the change in the peak breadths and levels of H3K14Ac and H3K23Ac are required for the alteration of gene expression in the presence of ethylene.

**Electronic supplementary material:**

The online version of this article (doi:10.1186/s12864-017-3929-6) contains supplementary material, which is available to authorized users.

## Background

Epigenomic modifications modulate gene expression in response to environmental biotic and abiotic stimuli and developmental cues [1–3]. For example, covalent modifications of histone proteins have been demonstrated to be essential gene expression modulators [4]. Histone modifications can be associated with chromatin-dependent transcriptional activation or repression depending on the specific amino acid substrate. Combined mutations at K5, K8, and K12 residues of histone H4 have significant impact on gene expression, whereas the individual mutations have negligible effects [5]. This combinations of various histone modifications is collectively known as the ‘histone code’ [6].

Histone acetylation counteracts the tendency of nucleosomal fiber that folds into a highly compact structure [7, 8], acetylated chromatin is more accessible to interacting proteins. The histone acetyltransferases and deacetylases that modify histone proteins provide a critical link between chromatin structure and transcriptional output [9]. Histone acetylation influences DNA replication, DNA repair, gene silencing, genome defense, genome organization, normal growth and development, aging, and numerous diseases [10–12]. Recently, histone acetylation has been implicated in responses of plants to hormones [13–15]. For example, abscisic acid (ABA) induces the elevation of acetylation at H3K9 and H3K14 in the promoter of *phas* gene, and the elevation is positively associated with gene expression [16]. MSI1 is a partner of the histone deacetylase HDA19 that fine-tunes ABA signaling by binding to the chromatin of ABA receptor genes to maintain a low level of histone acetylation at H3K9, thereby affecting the expression levels of ABA receptor genes [17]. Furthermore, HDA19 interacts with SNL1 and SNL2 to regulate acetylation of histone H3 at K9/18 and K14 in response to ABA [18].

A number of studies have focused on the role of histone acetylation in ethylene signaling [19–22]; however, how the genomic landscape of chromatin states is altered in response to ethylene and how histone acetylation integrates in the control of gene expression in response to ethylene signaling are not well characterized. Recently, we found that ethylene can specifically elevate histone H3 acetylation at K14 and at the non-canonical site K23 in etiolated seedlings, and the gene activation is positively associated with the elevations in H3K14Ac and H3K23Ac in response to ethylene [23]. In addition, we found that both EIN2 [24–26] and EIN3 [27] are involved.

Here, we present an in-depth epigenomic analysis of acetylation of H3K9, H3K14, and H3K23 in both wild-type *Arabidopsis* and the *ein2-5* mutant, which is completely ethylene insensitive. In this study, we compared the global histone H3 acetylation patterns at K9, K14, and K23 in wild-type (Col-0) and *ein2-5*-mutant etiolated seedlings with or without ethylene gas treatment. We discovered that H3K9Ac, H3K14Ac and H3K23Ac preferentially mark highly expressed genes at transcription start site (TSS) regions both in Col-0 and *ein2-5* mutant seedlings. Neither heights nor breadths of H3K9Ac ChIP peaks were altered by ethylene, but differences in H3K9Ac peak heights and breadths before ethylene treatment distinguish ethylene up- and down- regulated genes, and changes in H3K14Ac or H3K23Ac are observed in genes that responding to ethylene treatment. Overall, our study revealed ethylene induces combinatorial effects of H3K9Ac, K14Ac and K23Ac histone acetylation in gene expression genome widely. Further, for a group of ethylene regulated genes, in the absence of ethylene, the levels and the peak breadths of H3K9Ac are the potential preexist markers for distinguishing up- and down- regulated genes; in the presence of ethylene, the changes in the peak breadths and levels of H3K14Ac and H3K23Ac are required for the alteration of gene expression.

## Methods

### Plant growth conditions

*Arabidopsis* seeds were surface-sterilized in 50% bleach with 0.01% Triton X-100 for 15 min and washed five times with sterile, doubly distilled H_2_O before plating on MS medium (4.3 g MS salt, 10 g sucrose, pH 5.7, 8 g phyto agar per liter). After 3-4 days of cold (4 °C) treatment, the plates were wrapped in foil and kept in at 24 °C in an incubator before the phenotypes of seedlings were analyzed. For propagation, seedlings were transferred from plates to soil (Pro-mix-HP) and grown to maturity at 22 °C under 16-h light/8-h dark cycles. Ethylene treatment of *Arabidopsis* seedlings was performed by growth of seedlings on MS plates in air-tight containers in the dark supplied with either a flow of hydrocarbon-free air (Zero grade air, AirGas) or hydrocarbon-free air with 10 ppm (ppm) ethylene as previously described [28].

### Chromatin immunoprecipitation (ChIP) assays

ChIP was performed according to a published protocol [29, 30]. Briefly, 3-day-old etiolated seedlings treated with air or ethylene were harvested and crosslinked in 1% formaldehyde, and then ground into fine power with liquid nitrogen. Chromatin was isolated and sheared into 200–800 base pair fragments by sonication. Antibody H3K9Ac (Millipore; 07-352, 1:3000 dilution), anti-H3K14Ac (Millipore; 07-353, 1:2000 dilution), anti-H3K23Ac (Millipore; 07-355, 1:3000 dilution), and Magnetic Protein G Beads (Promega, G747A) were added to the sonicated chromatin followed by incubation overnight to precipitate bound DNA fragments. In this work, 2 mg antibody per reaction was used. The precipitated DNA was then recovered and ChIP-qPCR was performed with three technical replicates, and results were calculated as percentage of input DNA. Independent ChIP experiments were performed at least two more times and similar results were obtained. Primer sequences used for ChIP-qPCR were listed in Additional file 2: Table S1.

### ChIP-seq

Ten ng of ChIP DNA immuoprecipitated by antibody was used for ChIP-seq library construction. End repair, adapter ligation and amplification were carried out using NEB ChIP-seq Library Kit (NEB #E6240S/L) according to the manufacturer’s protocol. HiSeq 2000 (Illumina) was used for high-throughput sequencing of the ChIP-seq library in the UW-Madison Biotechnology Center. Independent ChIP-seq experiments, as well as IgG control, were performed two times.

### ChIP-seq data analysis

Raw ChIP-seq data from a previous study [23] were downloaded from NCBI GEO under GSE77396. H3K9Ac ChIP data for *ein2-5* seedlings under air and ethylene treatment were collected for this study. Initial quality-control analysis was performed using FastQC [31]. Single-end 51-bp reads were mapped to the *Arabidopsis* genome (TAIR10) [32] and uniquely mapped sequencing reads were generated using bowtie software (version 1.1.2) [33, 34]. For each histone modification in each condition, mapped reads were pooled across ChIP-seq replicates as described [23, 35]. Peaks significantly enriched in ChIP-seq tags were identified by Model-based Analysis for ChIP-Seq (MACS2, version 2.1.0.20150603; parameters: --nomodel,-p 0.01) as previously described [36]. Differential peaks were identified using the MAnorm method [37]. For this method, the normalized M value (M = log2 (Read density in C_2_H_4_ treated sample/Read density in air treated sample)) represents log2-transformed fold changes of enrichment intensities at each peak region [37–39]. Thus, an absolute threshold value of M ≥ 0.4 and *P* ≤ 0.05 were used to select differentially enriched peaks as done previously [23]. The nearest gene was assigned if there was more than one gene within 5 kilobases (kb) of the peak region [29]. Biological functions of associated genes were assessed by agriGO [40]. To show ChIP binding signal surrounding TSSs or in gene bodies, read coverage was first calculated using the bamCompare tool (RPKM, Log2(ChIP/IgG) in deepTools version 1.5.11 [41]. Heat maps of ChIP-seq enrichment across gene body regions were calculated using deepTools version 1.5.11 [41]. Generation of dot plots, heat maps (in Fig. 2b, Fig. 6 c-e), venn diagrams and statistical analyses were performed using R (version 3.2.2).

### RNA-seq data analysis

RNA-seq raw data were downloaded from NCBI GEO under GSE77396 [23, 25]. Initial quality-control analysis was performed using FastQC [31]. Raw reads were aligned to TAIR10 genome release using TopHat version 2.0.9 [42] with default parameters. Differential expressed genes were identified using Cufflinks version 2.2.1 following the workflow with default parameters [43]. Gene expression levels (RPKM, Reads Per Kilobase per Million mapped reads) in air and ethylene condition were generated from the output files of cuffdiff [43]. Differentially expressed genes were those for which relative fold change values (RPKM) of larger than 1.5 and RPKM value larger than 1 were observed [44].

### Real-time PCR

Total RNA was extracted using a Qiagen Plant Total RNA Kit (Sigma) from 3-day-etiolated seedlings treated with air or 4 h ethylene gas. First-strand cDNA was synthesized using Invitrogen Superscript III First-Strand cDNA Synthesis Kit. PCR reactions were performed in a total volume of 20 mL, containing 2 mL each 5-mM primer and 10-mL SYBR Green PCR Supermix in triplicate on a Roche 96 Thermal cycler according to the manufacturer’s instruction. The cycling program comprised an initial denaturation step at 95 °C for 10 min, followed by 50 cycles of 95 °C for 10 s, 60 °C for 10 s, and 72 °C for 20 s. All qRT-PCR values were normalized using the cycle threshold value corresponding to the reference gene. The relative expression levels of the target gene were calculated using 2(−Delta Delta C(T)) method [45]. The sequences of all primers are listed in Additional file 2: Table S1.

## Results

### Genome-wide mapping of H3K9, H3K14, and H3K23 in wild-type etiolated seedlings

**Fig. 1:**
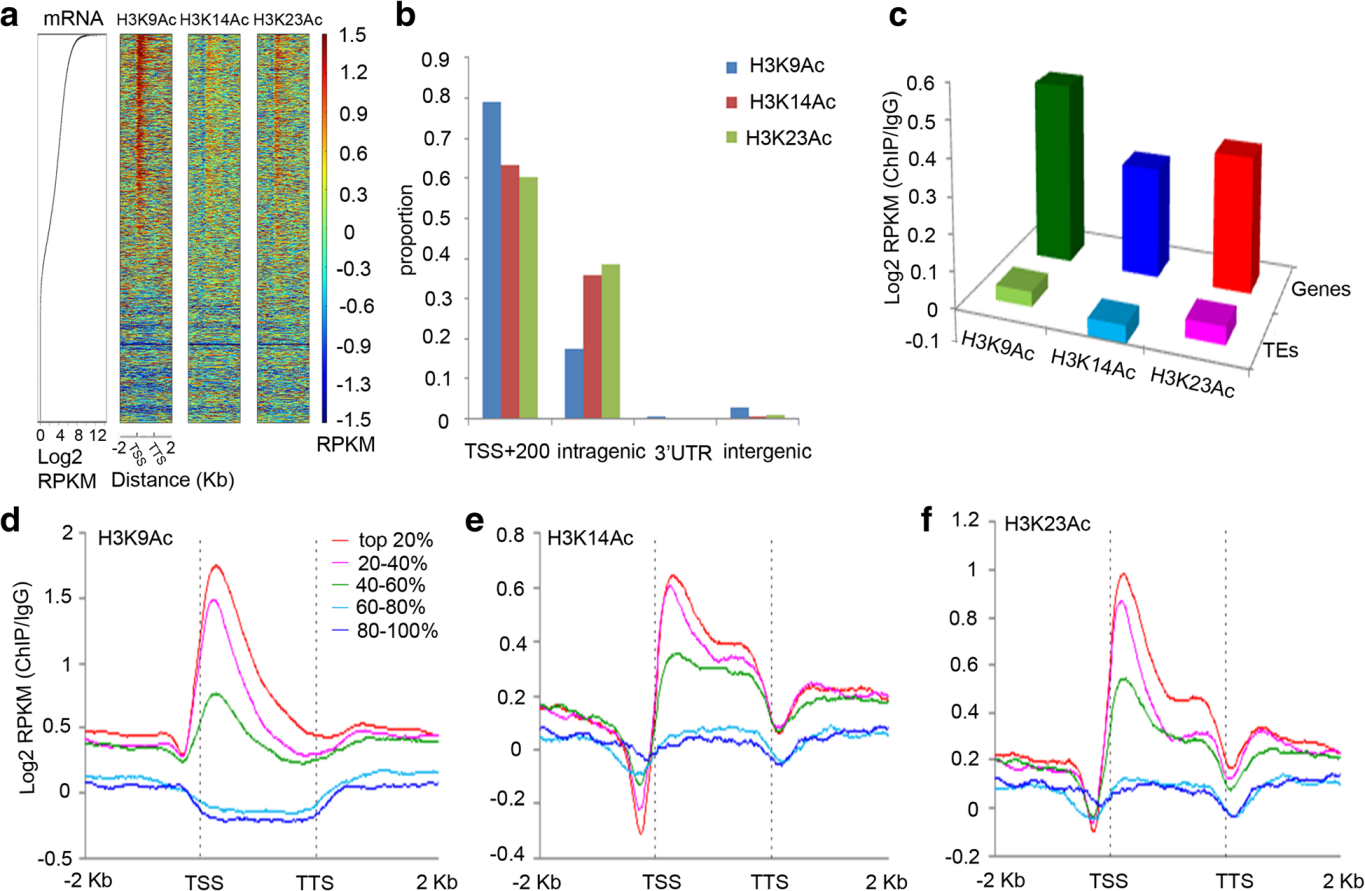
Histone acetylation marks are enriched around transcription start sites (TSS) and are positively correlated with gene expression levels in *Arabidopsis*. **a** Heat maps show H3K9Ac, H3K14Ac, and H3K23Ac enrichment (IgG normalized reads per kilobase (kb) per million mapped reads (RPKM)) at all genes in *Arabidopsis* along with relative mRNA values. Scatter plot for mRNAs ranked according to gene expression levels in 3-day-old Col-0 seedlings under air treatment. Heat maps are ranked according to gene expression levels. **b** H3K9Ac, H3K14Ac, and H3K23Ac peak distributions at the TSS and 200 bp downstream of the TSS (TSS + 200 bp), in intragenic regions (inside exons or introns excluding the peaks that overlapped with the TSS + 200 bp and 3′ UTR), in 3′ UTRs, and in intergenic regions (upstream and downstream of genes). **c** H3K9Ac, H3K14Ac, and H3K23Ac were highly enriched in genes compared to transposable elements (TEs). IgG normalized RPKM in gene body from the TSS to the transcription termination site (TTS) for each histone mark was calculated in all genes and for TEs. **d**-**f** Mean enrichment profiles (Log2 RPKM (ChIP/IgG)) along gene bodies of reads for **d** H3K9Ac, **e** H3K14Ac, and **f** H3K23Ac. Genes were ranked according to relative mRNA expression levels and divided into five equal sets

Our previous study showed that H3K14Ac and H3K23Ac but not H3K9Ac are specifically elevated by ethylene [23]. To fully understand the function of H3K9Ac, H3K14Ac, and H3K23Ac, we re-analyzed the ChIP-seq data from our previously published data obtained from 3-day-old etiolated Col-0 seedlings (Additional file 1: Figure S1 a,b) [23]. ChIP-seq profiles revealed that all three histone acetylation marks are enriched in promoters and intragenic regions (Fig. 1a and b). Furthermore, the three acetylation marks were depleted in transposable elements (Fig. 1c). To further examine the correlation between gene expression and three histone acetylation marks, we divided the approximately 30,000 genes in the *Arabidopsis* genome into five groups based on expression levels in wild type plants (Additional file 1: Figure S1c). We found that highly expressed genes show significantly higher levels of H3K9Ac, H3K14Ac, and H3K23Ac both near TSS regions and in gene bodies than the genes expressed at low levels (Fig. 1a, d-f).

### H3K9Ac, not the combined enrichment of H3K9Ac, H3K14Ac, and H3K23Ac, is critical for gene expression in the absence of ethylene treatment

**Fig. 2:**
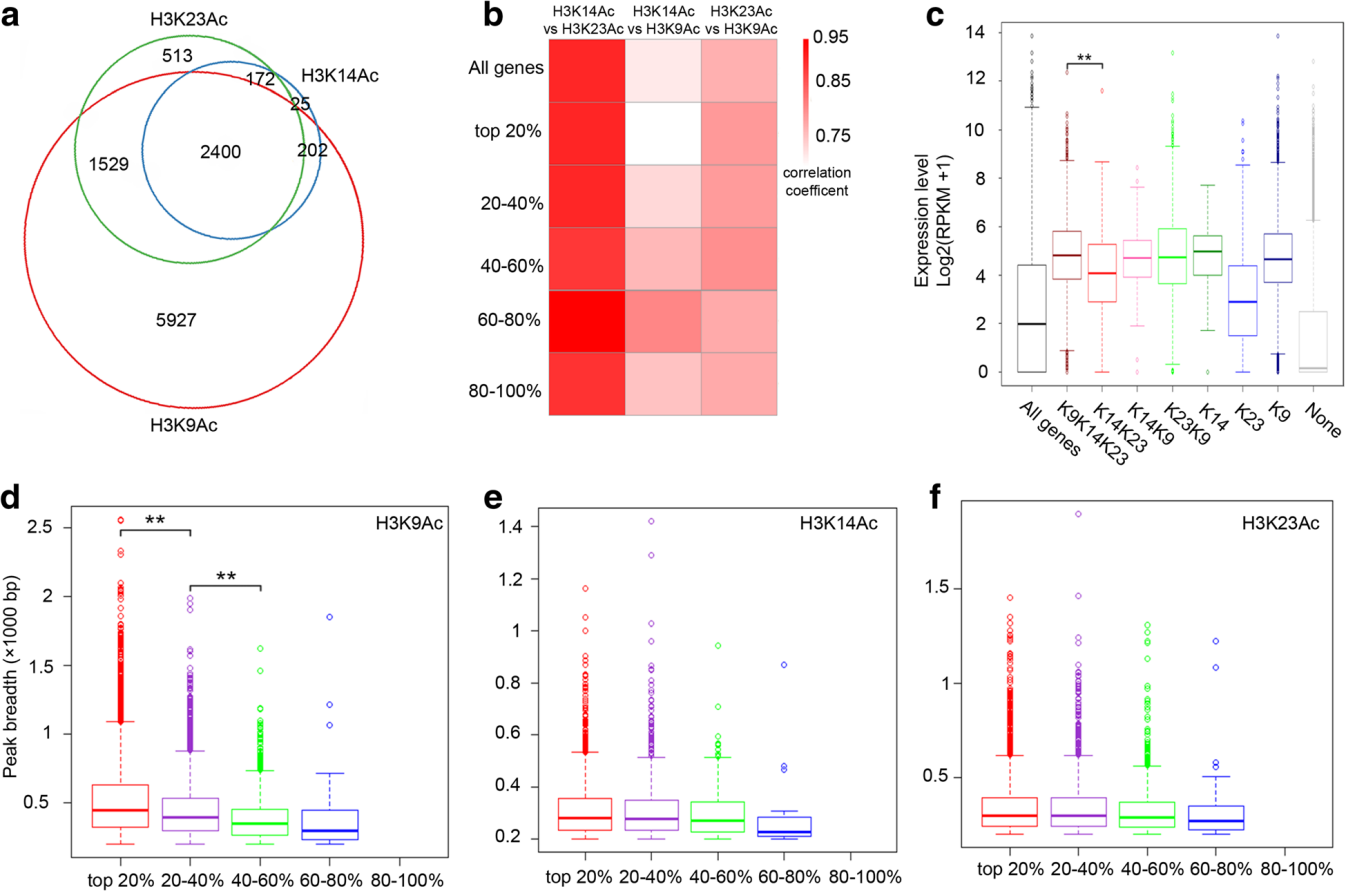
Genome-wide analysis of H3K9Ac, H3K14Ac and H3K23Ac in etiolated seedlings in the absence of ethylene. **a** Overlap of peak-associated genes among H3K9Ac, H3K14Ac and H3K23Ac in Col-0 under air treatment. The nearest gene was assigned to each peak if there was more than one gene within 5 kb. **b** The correlation of histone enrichment (RPKM) of H3K14Ac vs. H3K23Ac; H3K14Ac vs. H3K9Ac; and H3K23Ac vs. H3K9Ac in gene body regions with transcript abundance in the absence of ethylene. “All genes” indicates the total genes in *Arabidopsis*. Genes were ranked according to relative mRNA expression levels and divided into five equal sets. **c** Boxplot showing the association of transcript levels of gene expression and histone acetylation in Col-0 without ethylene treatment. Gene expression level was Log2-transformed. “All genes” indicates the total genes in *Arabidopsis*; K9 K14 K23, genes marked by H3K9Ac, H3K14Ac and H3K23Ac, *n* = 2400; K14 K23, genes specifically marked by H3K14Ac and H3K23Ac but not H3K9Ac, *n* = 172; K14 K9, genes specifically marked by H3K14Ac and H3K9Ac but not H3K23Ac, *n* = 202; K23 K9, genes specifically labeled by H3K23Ac and H3K9Ac but not H3K14Ac, *n* = 1529; K14, K23, and K9, peak associated genes uniquely marked by K14 (*n* = 25), K23 (*n* = 513), and K9 (*n* = 5927), respectively; none indicates genes not associated with H3K9Ac, H3K14Ac, or H3K23Ac peaks. The ** indicates *P* = 5.49 × 10^−11^ by t-test. **d**-**f** Boxplots showing the correlation of peak breadths for H3K9Ac (**d**), H3K14Ac (**e**), and H3K23Ac (**f**) with gene expression levels in genome wide. Genes were ranked according to relative mRNA expression levels and divided into five equal sets. The ** indicates *P* ≤ 3.25 × 10^−18^ by t-test. No significant differences within gene groups were detected between H3K14Ac and H3K23Ac gene sets (*P* value >0.01)

We found that about 92% of H3K14Ac-marked genes were overlapped with genes marked with H3K23Ac. In addition, 85% of H3K23Ac-marked genes were also marked with H3K9Ac, and about 93% of H3K14Ac-marked genes overlapped with H3K9Ac-marked genes (Fig. 2a, Additional file 1: Figure S2a and Additional file 2 Table S2). Thus, H3K14Ac and H3K23Ac preferentially co-exist with H3K9Ac. In pairwise association analyses we found that H3K9Ac is positively correlated with both H3K14Ac and H3K23Ac (Fig. 2b). Interestingly, the correlation between H3K14Ac and H3K23Ac is more significant than the others pairs (Fig. 2b, Pearson’s correlation coefficient = 0.93, *P* < 2.2 × 10^−16^).

To further explore how these three histone marks coordinate to regulate gene expression, we divided genes marked by H3K9Ac, H3K14Ac, or H3K23Ac into seven clusters based on the distributions of three histone marks (Fig. 2a, Additional file 1: Figure S2a and Additional file 2 Table S2). Approximately 2400 genes were marked by all three histone modifications, 202 genes were marked with both H3K9Ac and H3K14Ac but not H3K23Ac, 1529 genes contained both H3K9Ac and H3K23Ac but not H3K14Ac, and 172 genes were marked with H3K14Ac and H3K23Ac but not H3K9Ac. Only 25 genes were uniquely marked with H3K14Ac, and 513 genes were uniquely marked with the H3K23Ac mark, indicating that H3K14Ac and H3K23Ac preferentially co-exist with each other or with H3K9Ac (Fig. 2a, Additional file 1: Figure S2a and Additional file 2 Table S2).

By comparing transcript abundance within different clusters of genes, we found that the genes without histone marks were expressed at the lowest levels (Fig. 2c), which is consistent with previous studies [46]. Interestingly, the expression levels of the genes containing all three histone marks were not significant higher than that of the genes containing H3K9ac/H3K14ac, H3K9ac/H3K23Ac, or containing only H3K14Ac, or H3K9Ac (K9/K14/K23 vs. K14/K23, *P* = 5.49 × 10^−11^; K9/K14/K23 vs. K14/K9, *P* = 0.154; K9/K14/K23 vs. K9/K23, *P* = 0.171). In addition, expression levels of the genes containing only with H3K23Ac were lower than that of the genes in any other clusters. Notably, genes in any all clusters containing H3K9Ac showed similar gene expression levels (Fig. 2c). In order to confirm the observation, we did a similar analysis using the top 20% enriched peaks associated genes, and similar results were obtained (Additional file 1: Figure S2b, c), indicating that H3K9Ac is involved in the determination of genome-wide gene expression levels in etiolated seedlings.

We next compared the peak breadths for each of the three histone marks to mRNA transcript levels. We found peak breadths in the ChIP data for histone H3K9Ac were positively correlated with gene expression in the genes at the highest three levels of transcript abundance, whereas there were no correlations between the peak breadths of H3K14Ac or H3K23Ac with gene expression in the absence of ethylene (Fig. 2d-f). These results revealed a positive correlation between transcript abundance and levels of H3K9Ac, H3K14Ac, and H3K23Ac.

### Ethylene induces combinatorial effects of H3K9Ac, H3K14Ac, and H3K23Ac in gene expression

**Fig. 3:**
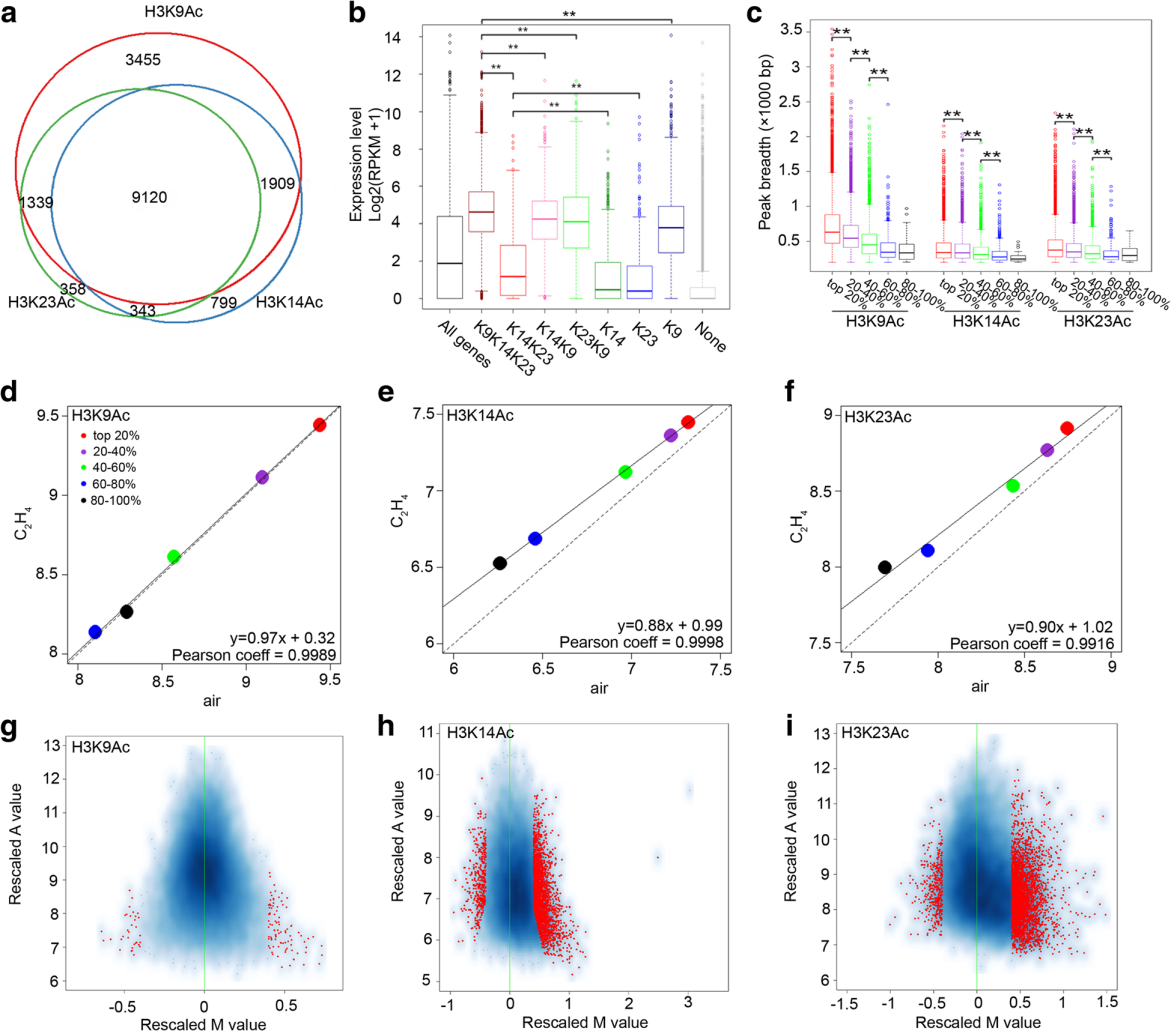
Combined enrichment of H3K9Ac, H3K14Ac, and H3K23Ac is correlated with high gene expression levels in the presence of ethylene. **a** Overlap of genes marked by H3K9Ac, H3K14Ac and H3K23Ac under ethylene treatment. **b** Boxplot showing the association of transcript levels of gene expression and histone acetylation in Col-0 with ethylene treatment Gene expression levels were Log2-transformed. “All genes” indicates the total genes in *Arabidopsis*; K9 K14 K23, genes marked by H3K9Ac, H3K14Ac and H3K23Ac, *n* = 9120; K14 K23, genes marked by H3K14Ac and H3K23Ac but not H3K9Ac, *n* = 343; K14 K9, genes marked by H3K14Ac and H3K9Ac but not H3K23Ac, *n* = 1909; K23 K9, genes marked by H3K23Ac and H3K9Ac but not H3K14Ac, *n* = 1339; K14, K23, and K9, genes uniquely marked by K14 (*n* = 799), K23 (*n* = 358), or K9 (*n* = 3455); none, genes that could not be assigned to peaks of H3K9Ac, H3K14Ac, or H3K23Ac. The ** indicates *P* < 6.04 × 10^−6^ by t-test. **c** Boxplot showing peak breadths under ethylene treatment plotted based on transcript abundance. Genes were ranked according to relative mRNA expression levels and divided into five equal sets. The ** indicates *P* < 0.001 by t-test. **d**-**f** Scatter plots of mean histone enrichment of **d** H3K9Ac, **e** H3K14Ac, and **f** H3K23Ac in ethylene treatment vs. air. To generate the graph, histone reads density were normalized using MAnorm [37] and normalized peak enrichments in ethylene vs. air were plotted for sets of genes clustered based on transcript abundance in air. *Solid black lines* in the plots denote the linear regression line. **g**-**i** Volcano plots for **g** H3K9Ac, **h** H3K14Ac, and **i** H3K23Ac showing the differential enrichment (Rescaled M value versus Rescaled A value after MAnorm normalization [37]) for each individual peak detected in the comparison of plants grown in ethylene vs. air. M = log2 (Read density in C_2_H_4_/Read density in air) and A = 0.5 × log2 (Read density in C_2_H_4_ × Read density in air). Red dots represent data with *P* ≤ 0.05 and |M| ≥0.4

To study whether and how ethylene leads to the H3K14Ac, H3K9Ac and H3K23Ac changes genome wide, we examined the ChIP-seq profiles of H3K9Ac, H3K14Ac, and H3K23Ac in seedlings treated with ethylene. The ChIP-seq profiles of H3K9Ac and H3K23Ac were not significantly changed by ethylene (Additional file 1: Figure S3a). In the presence of ethylene, the three histone marks were more highly correlated (based on Pearson coefficient) than in the absence of ethylene with the largest increased in the association between H3K14Ac and H3K23Ac (Additional file 1: Figure S3b). To further explore how the three histone marks coordinate in regulation of gene expression in response to ethylene, we compared correlations within seven clusters of the genes divided based on transcript abundance (Fig. 3a and Additional file 2 Table S3). The abundances of the genes marked only with H3K14Ac or only with H3K23Ac were the lowest in all clusters, and no “biological process” GO terms were significantly associated with these genes. The genes marked by both H3K14Ac and H3K23Ac had higher expression levels than those marked with H3K14Ac only or H3K23Ac only (Fig. 3b) (the expression of genes marked by K14/23Ac vs. the expression of genes marked by K14Ac, *P* = 1.52 × 10^−7^; the expression of genes marked by K14/23Ac vs. the expression of genes marked by K23Ac, *P* = 6.04 × 10^−6^).

In addition, in the presence of ethylene, the genes with all three histone marks had significantly higher transcript abundances than other genes with one or two of the marks (Fig. 3b; K9/K14/K23 vs. K9/K14, *P* = 1.31 × 10^−26^; K9/K14/K23 vs. K9/K23, *P* = 3.29 × 10^−28^; K9/K14/K23 vs. K14/K23, *P* = 1.6 × 10^−176^; K9/K14/K23 vs. K9, *P* = 9.6 × 10^−138^; K9/K14/K23 vs. K14, *P* = 0; K9/K14/K23 vs. K23, *P* = 3.2 × 10^−270^). In the presence of ethylene, the breadths of ChIP peaks for all three histone marks showed a significant positive correlation with transcript abundance (Fig. 3c). These results reveal that H3K9Ac, H3K14Ac and H3K23Ac have combinatorial effects in gene expression.

To further compare the histone marks between with and without ethylene treatments, we checked histone enrichment in different sets of peak associated genes. We found that highly expressed genes had significantly higher levels of the H3K9Ac, H3K14Ac, and H3K23Ac both with and without ethylene treatments (Fig. 3d-f). The levels of both H3K14Ac and H3K23Ac, were elevated by ethylene, whereas no difference was detected in H3K9Ac levels (Fig. 3d-f), which is consistent with a previous report [23]. We then compared differential peaks induced by ethylene for all three histone marks. Significant differences in H3K14Ac and H3K23Ac were observed in the presence of ethylene relative to in the absence of ethylene, and fewer differential H3K9Ac peaks were identified (Fig. 3g-i). Thus, the combined histone enrichment is positively associated with high gene expression levels.

**Fig. 4:**
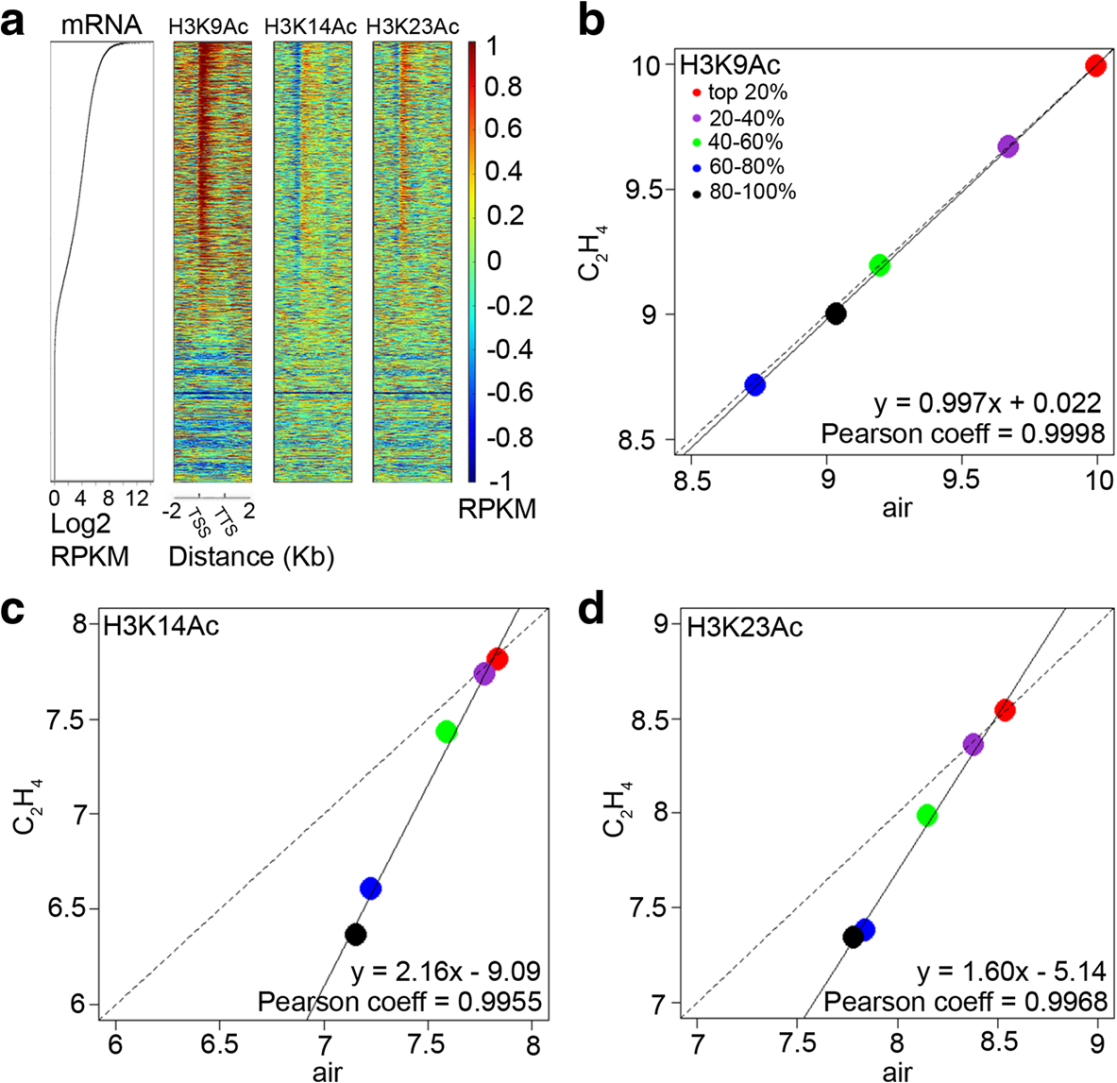
The ethylene-induced shift of histone acetylation marks is EIN2 dependent. **a** Heat maps show H3K9Ac, H3K14Ac and H3K23Ac enrichment (IgG normalized reads per kilobase per million mapped reads (RPKM)) in *ein2-5* at all genes in the *Arabidopsis* which along with relative mRNA values. Scatter plot for mRNAs was ranked according to gene expression levels in Col-0 under air treatment. Heat maps were ranked according to their gene expression levels. **b**-**d** Scatter plot shows mean histone enrichment of H3K9Ac (**b**), H3K14Ac (**c**) and H3K23Ac (**d**) in air versus ethylene treatment in *ein2-5*. To generate the graph, histone peaks were normalized by MAnorm [37], and peak-associated genes were ranked according to relative mRNA expression levels and divided into five equal sets. Then the normalized peak enrichments in air and ethylene condition were plotted. *Solid black lines* in the plots denote the linear regression line

To further explore the regulation of histone acetylation in response to ethylene, we did ChIP-seq of H3K9Ac in the *ein2-5* mutant, which is completely ethylene insensitive [23]. As in Col-0 seedlings, highly expressed genes in 3-day-old *ein2-5* seedlings had significantly higher levels of H3K9Ac, H3K14Ac, and H3K23Ac than all groups of genes both near the TSSs and in the gene bodies. Genes expressed at low levels had lower levels of these three histone marks across the entire gene body (Fig. 4a and Additional file 1: Figure S4a-c). Different from Col-0, in the *ein2-5* mutant, the levels of H3K9Ac were not altered by ethylene; however, H3K14Ac and H3K23Ac enrichment was reduced in genes in the three abundance groups, but not the two highest groups, in seedlings grown in ethylene compared to those grown in air (Fig. 4b-d). When Col-0 plants treated with ethylene, the ChIP peak breadths for all three histone marks showed significant positive correlations with gene expression genome wide (Fig. 3c). In *ein2-5* mutant seedlings, the ChIP signals showed significantly positive correlations with gene expression in both the presence and absence of ethylene (Additional file 1: Figure S4d and e); however, no ethylene-induced enhancements were detected, revealing that the ethylene-induced increase in acetylation of histone H3 is EIN2 dependent (Additional file 1: Figure S4d and e).

### The combined effects of H3K9Ac, H3K14Ac, and H3K23Ac on the expression of ethylene-regulated genes

**Fig. 5:**
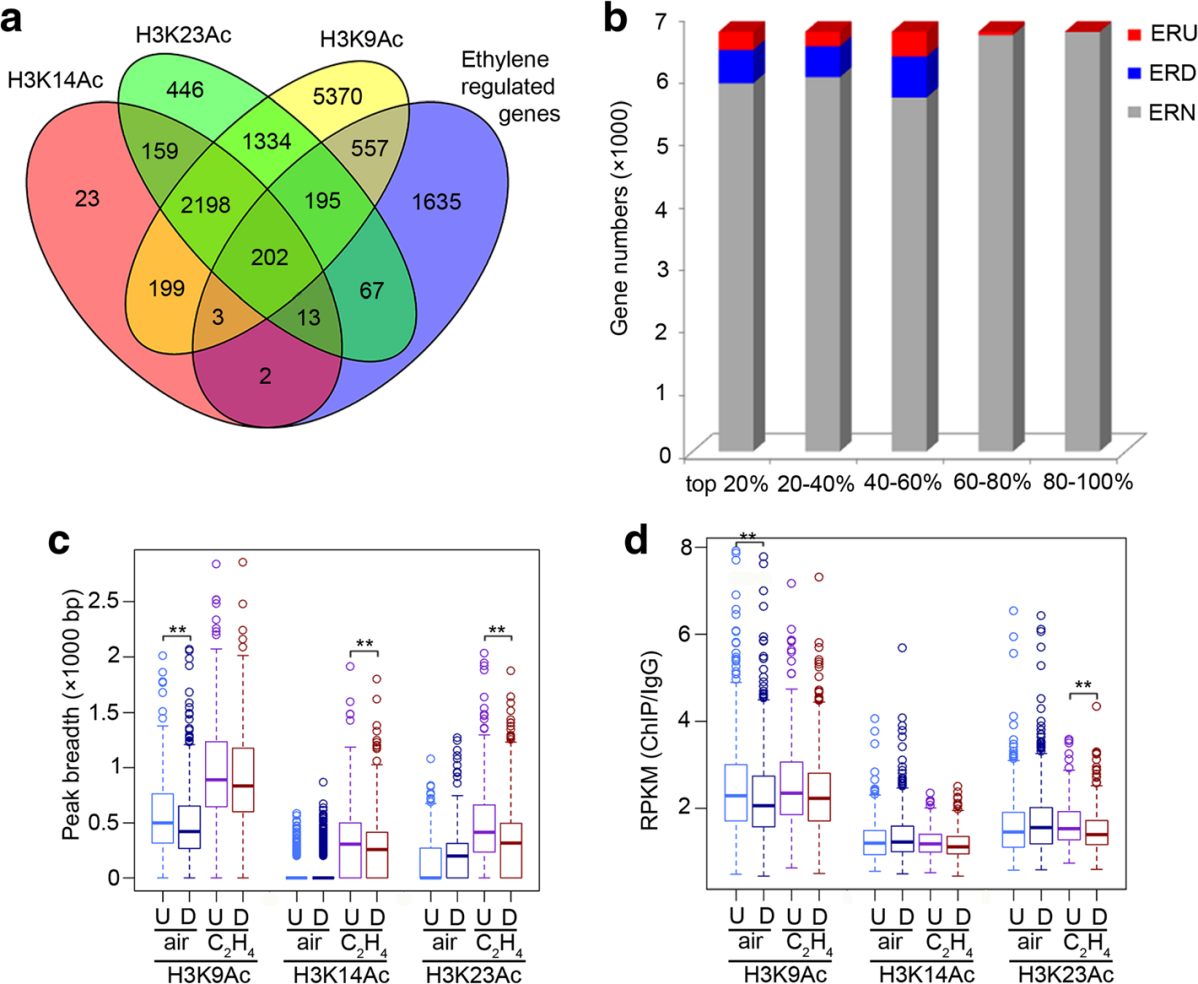
Histone acetylation patterns in ethylene-regulated genes. **a** Overlap of ethylene-regulated genes marked by H3K9Ac, H3K14Ac, and H3K23Ac in Col-0 under air treatment. **b** Numbers of *Arabidopsis* genes that are ethylene up-regulated (ERU), ethylene down-regulated (ERD), and not regulated by ethylene (ERN) based on relative mRNA expression levels divided into five equal sets. **c** Boxplot showing the correlation of peak breadths and ethylene up-regulated genes (U, n-405) or ethylene down-regulated genes (D, n-634) in COL-0 under air and ethylene treatment. The ** indicates *P* < 0.001 by t-test. **d** Boxplot showing the histone mark enrichment (RPKM) in 1000 bp around TSSs in ethylene up-regulated genes (U, n-405) and ethylene down-regulated genes (D, n-634) in Col-0 in air and ethylene. The ** indicates *P* < 0.001 by t-test

To further study how histone acetylation integrates into the regulation of gene expression, we re-analyzed the published RNAseq data from 3-day-etiolated seedlings treated with air or 4 h ethylene gas [23, 25], and further isolated the ethylene-regulated genes that are marked by any one of the three histone marks. In total 1039 genes were identified (Fig. 5a and Additional file 2 Table S4). In this group of genes, in the absence of ethylene, the enrichments of H3K14Ac and H3K23Ac were observed more often in intragenic than in promoter regions in the absence of ethylene (Additional file 1: Figure S5a-g). The opposite was the case in our genome-wide analysis (Fig. 1b). In the presence ethylene, there was no change in H3K9Ac in ethylene regulated genes (Additional file 1: Figure S5g); however, enrichment in the promoter regions and intragenic regions for H3K23Ac and H3K14ac were increased (Additional file 1: Figure S5d-g). The ethylene-regulated genes (total 2674) were mainly (97.8%) expressed at high or medium levels (Fig. 5b, Additional file 1: Figure S5a-f), indicating that certain levels of histone modification and gene expression above a certain threshold characterize a group of genes responding to ethylene. We then examined whether the combination of these three histone acetylation marks is associated with changes in gene expression in the presence of ethylene (Additional file 1: Figure S5 h-j and Additional file 2 Table S5). The genes were divided into seven clusters based on the combination of acetylation of H3. No significant correlations were detected between the differences in gene expression in the presence and absence of ethylene with any combined enrichments of the three histone acetylation marks (Additional file 1: Figure S5i-j).

We next divided ethylene-regulated genes marked by H3K9Ac, H3K14Ac, and/or H3K23Ac into two groups: ethylene-up regulated genes (*n* = 405) and ethylene-down regulated genes (*n* = 634) (Additional file 2: Table S4). In the absence of ethylene, the ChIP peak breadths for H3K9Ac were larger in ethylene up-regulated genes than in down-regulated genes, but no differences were detected for H3K14Ac and H3K23Ac (Fig. 5c). In the presence of ethylene, the peak breadths for each of the three histone marks increased in both up- and down-regulated genes. For both H3K14Ac and H3K23Ac, the peak breadths became larger in up-regulated genes than in down-regulated genes (Fig. 5c). Even though the H3K9Ac signal was not altered in ethylene (Fig. 3d) as reported previously [23], the levels enriched for H3K9Ac were higher in ethylene up-regulated genes than in ethylene down-regulated genes before ethylene treatment, and no significant difference was detected after ethylene treatment. Unlike H3K9Ac, the levels for H3K23Ac showed a positive association with gene expression after ethylene treatment, although not before ethylene treatment (Fig. 5d; Additional file 1: Figure S5 k,l). These results suggest that in the absence of ethylene, the H3K9Ac mark distinguishes ethylene up-regulated from ethylene down-regulated genes and that the changes in H3K14Ac and H3K23Ac are potentially required for the alteration of gene expression in response to ethylene.

**Fig. 6:**
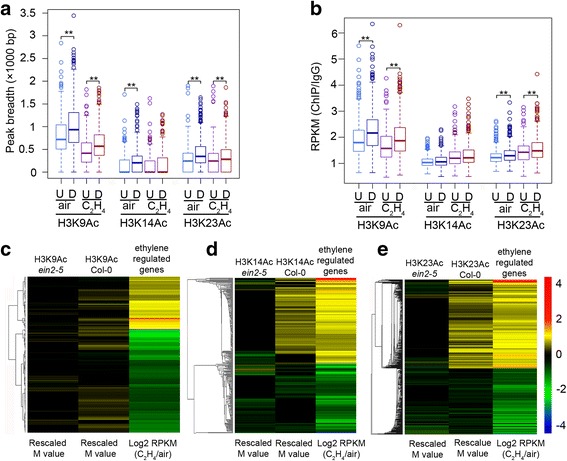
Histone acetylation in ethylene regulated genes in *ein2-5*. **a** Boxplot showing the peak breadths at their associated- ethylene regulated genes in *ein2-5* under air and ethylene treatment. “U” indicates peak associated-ethylene up regulated genes, *n* = 405; “D” indicates peak associated-ethylene down regulated genes, *n* = 634. The ** indicates *P* < 0.001 by t-test. **b** Boxplot showing the histone marks enrichment (RPKM) in 1000 bp around TSS at their associated- ethylene regulated genes in *ein2-5*. “U” indicates peak associated-ethylene up regulated genes, *n* = 405; “D” indicates peak associated-ethylene down regulated genes, *n* = 634. The ** indicates *P* < 0.001 by t-test. **c**-**e** Heat maps showing **c** H3K9Ac, **d** H3K14Ac, and **e** H3K23Ac differential enrichment (cutoff *P* ≤ 0.05) on genes differentially expressed in response to ethylene in Col-0 and *ein2-5* mutant seedlings. Only the genes marked by H3K9Ac, H3K14Ac, and H3K23Ac in Col-0 plants were used for this heat map. Each line in the heat map represents the representative histone-marked genes, first two lanes of the heat maps in c-e are the normalized M value (obtained using MAnorm, M = log2 (Read density in C_2_H_4_/Read density in air)) for differential peaks, and the last lanes of the heat maps in c-e are the fold changes of gene expression

Finally, we examined the histone acetylation of ethylene-regulated genes in the *ein2-5* mutant. Intriguingly, in *ein2-5* mutant, three histone marks showed a positive correlation with gene expression both with and without the presence of ethylene (Additional file 1: Figure S6a-f), which was similar to that of the Col-0 seedlings treated with ethylene, but the ethylene induced alterations in Col-0 were not detected in *ein2-5* mutant (Fig. 6a, b). Furthermore, in *ein2-5* mutant seedlings, the peak breadths in ethylene down-regulated genes were larger than those in ethylene up-regulated genes, and the signals of the three histone acetylation marks were also higher in ethylene down-regulated genes than in ethylene up-regulated genes both with and without ethylene (Fig. 6a, b). In addition, most transcripts from genes that were differentially marked by histone H3 acetylation in the presence versus the absence of ethylene were not detectable in *ein2-5* mutant (Fig. 6c-e). Collectively, the results show that EIN2 is required for the ethylene induced combinatorial effects of histone acetylation in a group of ethylene-regulated genes.

## Discussion

The correlation between histone acetylation and gene expression has been well studied [9, 47]. Here, we confirmed that H3K9Ac, H3K14Ac, and H3K23Ac are positively associated with gene expression levels genome-wide in *Arabidopsis* both with and without ethylene treatments (Fig. 1). A number of studies have shown that histone acetylation is involved in responses to plant hormones including ABA [17, 21, 48], jasmonic acid [19, 49], auxin [50, 51], and ethylene [19–22]. Little is known, however, about how histone acetylation alters in response to plant hormones genome-wide. Our study represents an important initial step toward a description of the genomic landscape of H3K9Ac, H3K14Ac, and H3K23Ac in response to ethylene. Many acetylated residues act coordinately and the combined effect ultimately dictates the functional outcome [52]. For example, acetylation at K5, K8, and K12 of H4 have redundant influences on nucleosome assembly and transcription [53]. Effects can also be combinatorial; for example, the combined accumulation of H3K23Ac and H4K16Ac is positively associated with gene expression in green plants [46]. Interestingly, we found that in the absence of ethylene, no combinatorial effect of histone acetylation on genome-wide gene expression was detected in 3-day-old etiolated Col-0 seedlings (Fig. 2c). However, in the presence of ethylene, a significant positive correlation between the combined labels of histone acetylation and high levels of gene expression was detected (Fig. 3b). In addition, when seedlings treated with ethylene, there was a positive correlation between H3K14Ac and H3K23Ac signals with the levels of gene expression. Acetylation of histones H3 and H4 leads to partial decondensation of chromosomal domains, although this opening is not necessarily tightly correlated with active transcription [54]. Our observation of an ethylene-induced positive association between H3K14Ac and H3K23Ac and gene expression is likely due to the combined effects of both an increase in the number of bases of the gene covered by H3K14Ac and H3K23Ac and an elevation in acetylation.

Genetic and biochemical studies suggest that histone acetyltransferases have rather specific roles in gene activation [55, 56], whereas genome-wide experiments suggest that several acetyltransferases are often recruited simultaneously and together acetylate multiple lysine residues at a given loci. Thus, the biological function of histone acetylation may be additive than highly specific. We found that in seedlings grown in the presence or absence of ethylene, H3K9Ac, H3K14Ac, and H3K23Ac coexist and the Pearson correlation between H3K9Ac and H3K23Ac is higher in ethylene-treated plants than those grown in air (Fig. 2b and Additional file 1: Figure S3b). One possibility is that ethylene treatment induces changes in the behaviors of histone acetyltransferases or histone deacetylates, leading to a tighter association of H3K9Ac and H3K23Ac. Alternatively, ethylene may cause changes in other histone modifications or effectors of chromatin configuration that lead to a tighter association between H3K9Ac and H3K23Ac.

The ethylene-induced changes in histone acetylation depended on EIN2. In our genome-wide analysis, the distributions of H3K9Ac, H3K14Ac, and H3K23Ac in the *ein2-5* mutant and in the Col-0 seedlings were very similar, all of these marks are preferentially enriched at moderately to highly expressed genes in *Arabidopsis,* consistent with the fact that acetylation marks are generally associated with open chromatin and actively transcribed regions [46, 57]. In wild-type plants, there are clear differences in the histone marks genome-wide between air and ethylene treatments (Fig. 2d-f, Fig. 3c), but in the *ein2-5* mutant, this shift was not detected (Additional file 1: Figure S4d and e). Most interestingly, the pattern in wild-type plants treated with ethylene was similar to that of the pattern in the *ein2-5* mutant, which indicates that EIN2 is important for maintenance of chromatin signatures and also important for the changes in histone acetylation in response to ethylene. In the presence of ethylene, EIN2 is up-regulated in wild-type plants, whereas EIN2 is absent in *ein2-5* mutant, why histone acetylation was similar between wild-type plants and *ein2-5* mutant is unclear. One possibility is that the levels of EIN2 fine tune the chromatin state. It will be important to determine at the molecular level how EIN2 regulates histone acetylation in the ethylene response.

The majority of ethylene-regulated genes are expressed at medium to high levels (Fig. 5b). That very few ethylene-regulated transcripts are low abundance, suggests that histone acetylation and a threshold of the expression level are potentially required for the genes to respond to ethylene treatment. Most interestingly, even though H3K9Ac is not regulated by ethylene, the enrichment of H3K9Ac in the ethylene up-regulated genes is higher than in the ethylene down-regulated genes (Fig. 5c). This difference was not observed for H3K14Ac and H3K23Ac signals (Fig. 5c). This suggests that in the absence of ethylene, the levels of H3K9Ac are important to determine the condensation of chromatin in activation regions or repression regions for ethylene response. In the presence of ethylene, the H3K14Ac and H3K23Ac are regulated by ethylene and positively correlated to gene expression in its presence.

How H3K9Ac enrichment is established is unknown. H3K9Ac and H3K4me3 are observed in highly expressed genes [56], and the locations and distributions of H3K9Ac and H3K4me3 influence transcriptional activity and expression in response to developmental and environmental changes. Chromatin regions with H3K4me3 are targeted for histone acetylation by histone acetyltransferases [56]. Therefore, one possible reason for the difference of H3K9Ac in the genes differentially regulated by ethylene is the preexistence of other histone marks, such as H3K4me3 or H3K27me3, that influence H3K9Ac levels. Alternatively, DNA sequences or the promoter structures may be different in ethylene up-regulated genes than in ethylene down-regulated genes, which leads to different levels of H3K9Ac.

The bromodomain, a evolutionarily conserved protein interacting module, has been reported to be responsible for acetylated lysine residues recognition [58]. The *Arabidopsis* proteome encodes 29 bromodomain-containing proteins [59]. GCN5/HAG1, a bromodomain-containing histone acetyltransferase that is required for the binding of GCN5 to a subset of its target chromatin loci [60], has been studied to be involved into many plant development processes, as well as environmental stimuli responses [61]. Rice OsHAG702, which shows high similarities with AtHAG1, was significantly elevated in response to exogenous ABA application to seedlings, indicating that it may be involved in the ABA signaling pathway for response to environmental stresses [62]. Some bromodomain proteins exhibit an extra terminal (ET) domain besides the N-terminal bromodomain(s), and they are named as BET proteins [59]. The BET domain functions as a protein-protein interaction domain to recruit other proteins to acetylated histones [63]. Twelve BET-encoding genes have been identified in the *Arabidopsis* genome, and some of are known to involve in plant development, mitotic cell cycle regulation and ABA response [64–67]. Considering the histone acetylation changes in response to ethylene [23], further studies are needed to confirm whether these bromodomain-containing proteins are involved in the ethylene response.

## Conclusions

Histone acetylation and deacetylation are essential for gene regulation and have been implicated in the regulation of plant hormone responses. Many studies have indicated the role of histone acetylation in ethylene signaling; however, few studies have investigated how ethylene signaling regulates the genomic landscape of chromatin states. This study reveals that the plant hormone ethylene induces combinatorial effects of H3K9Ac, K14Ac and K23Ac histone acetylation in gene expression both genome widely and a group of ethylene regulated genes, providing an insight knowledge of plant hormone induced combinatorial effect in gene expression.

## Additional files

Additional file 1: Figure S1. Gene expressions in *Arabidopsis*. **Figure S2.** Combined enrichment of H3K9Ac, H3K14Ac and H3K23Ac is correlated with high gene expression levels. **Figure S3.** Histone acetylation of H3K9Ac, H3K14Ac and H3K23Ac in response to ethylene. **Figure S4**. The ethylene induced shift of histone acetylation marks is EIN2 dependent. **Figure S5**. Histone modification in ethylene regulated genes. **Figure S6**. Histone modification in ethylene regulated genes in *ein2-5*. (PDF 1200 kb)

Additional file 2: Table S1. Primers for ChIP-qPCR and qPCR. **Table S2.** list of genome wide genes with differential histone markers in seedlings treated with air. **Table S3.** list of genome wide genes with differential histone markers in seedlings treated with ethylene. **Table S4.** list of ethylene regulated genes with differential histone markers in seedlings treated with air. **Table S5.** List of ethylene regulated genes with differential histone markers in seedlings treated with ethylene. (ZIP 1241 kb)

## Abbreviations

ABA: abscisic acid
ChIP-seq: Chromatin immunoprecipitation sequencing
EIN2: ethylene-insensitive protein 2
ENAP1: EIN2 nuclear associated protein 1
GO: Gene ontology
H3K14Ac: H3 lysine 14 (H3K14) acetylation
H3K23Ac: H3 lysine 23 (H3K23) acetylation
H3K9Ac: H3 lysine 9 (H3K9) acetylation
MS: Murashige & Skoog
RNA-seq: RNA sequencing
RPKM: Reads per kilo base per million mapped reads
