# Supplementary material for: Ethylene induces combinatorial effects of histone H3 acetylation in gene expression in Arabidopsis
Source: BMC Genomics. 2017 Jul 17;18:538. doi: 10.1186/s12864-017-3929-6 (PMC5512946; doi:10.1186/s12864-017-3929-6)
Supplement: Supplementary file 1 — Gene expressions in Arabidopsis. Figure S2. Combined enrichment of H3K9Ac, H3K14Ac and H3K23Ac is correlated with high gene expression levels. Figure S3. Histone acetylation of H3K9Ac, H3K14Ac and H3K23Ac in response to ethylene. Figure S4. The ethylene induced shift of histone acetylation marks is EIN2 dependent. Figure S5. Histone modification in ethylene regulated genes. Figure S6. Histone modification in ethylene regulated genes in ein2-5. (PDF 1200 kb) [file 12864_2017_3929_MOESM1_ESM.pdf]

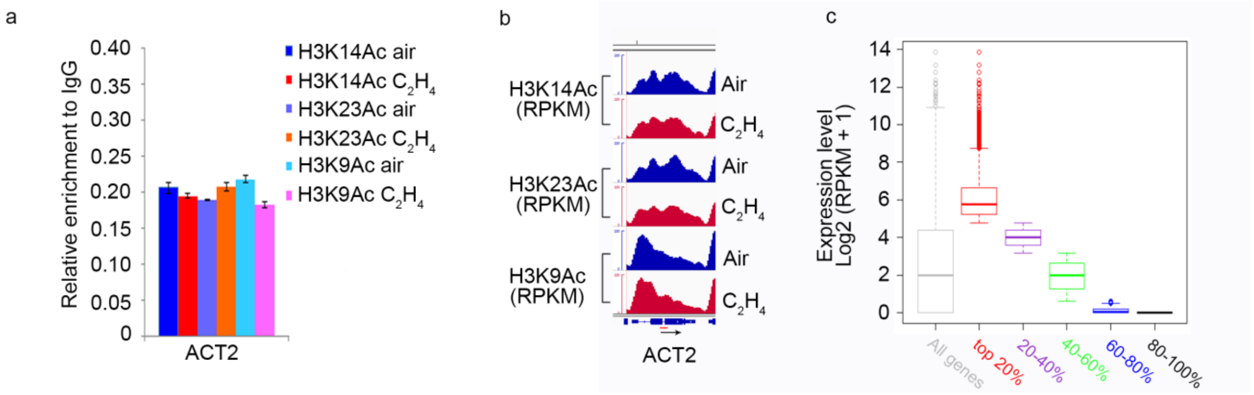

Additional file 1: Figure S1. Gene expressions in *Arabidopsis*. (a) ChIP quantitative real time PCR detection of H3K9Ac, H3K14Ac and H3K23Ac enrichment on ACT2 gene in Col-0 3-day-old etiolated seedlings treated with air or 4 hours ethylene gas. Precipitation with IgG pre-immune serum served as a control. Data represent the percentage of ChIP enrichment relative to IgG. (b) Standard ChIP-seq assays showing that the enrichment of H3K9Ac, H3K14Ac and H3K23Ac in ACT2 gene. Binding levels are indicated by reads per kilobase per million reads in sample (RPKM). Col-0 seedlings grown in the dark for 3 days with or without 4 hours ethylene gas treatment for ChIP-seq. Arrow indicates the direction of gene. Red line below the gene model indicates the ChIP-qPCR amplification region in (a). (c) Boxplot showing the Log2-transformed expression levels of different gene sets. Genes were ranked according to relative mRNA expression levels and divided into five equal sets.

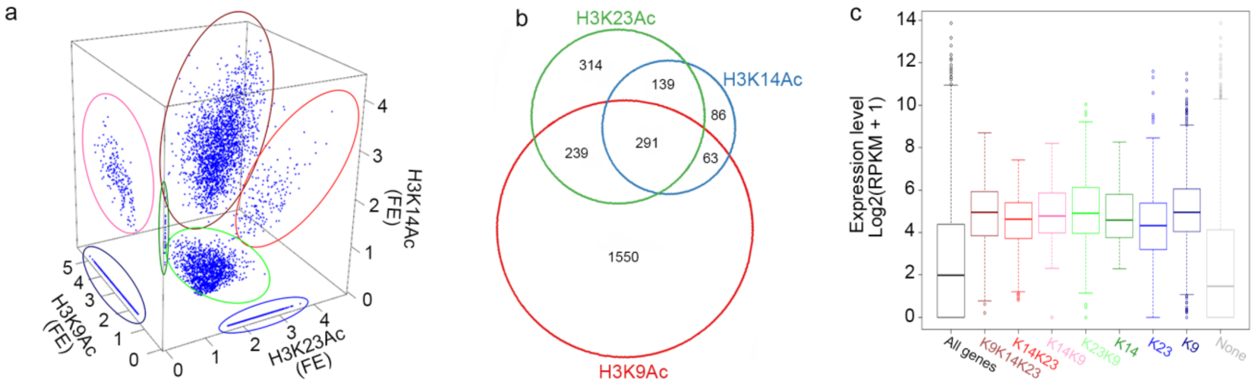

Additional file 1: Figure S2. Combined enrichment of H3K9Ac, H3K14Ac and H3K23Ac is correlated with high gene expression levels. (a) 3D plot shows the associations of H3K9Ac, H3K14Ac and H3K23Ac in different genes in Col-0 under air treatment. 7 gene clusters were divided based on the different combined labels of H3K9ac, H3K14ac and H3K23ac and histone levels. The colors for ellipses are the same as the boxplots respectively in Fig2c, indicating using the same set of peak associated genes. (b) Venn diagram showing the overlap of genes labeled by top 20% enriched peaks from each histone marks indicated in the figure, peaks were ranked according to fold-enrichment and top 20% of them were used for analysis. (c) Boxplot showing the correlation of gene expression and histone acetylation for the top 20% enriched peak associated genes. All genes indicates the total genes in *Arabidopsis*; K9K14K23, the overlapped peak associated genes among H3K9Ac, H3K14Ac and H3K23Ac, n=291; K14K23, peak associated genes specifically labeled by H3K14Ac and H3K23Ac, but not H3K9Ac, n=139; K14K9, peak associated genes specifically labeled by H3K14Ac and H3K9Ac, but not H3K23Ac, n=63; K23K9, peak associated genes specifically labeled by H3K23Ac and H3K9Ac, but not H3K14Ac, n=239; K9, K14 and K23, peak associated genes uniquely labeled by K9 (n=1550), K14 (n=86), K23 (n=314) separately; none, genes that could not be assigned to any peaks of H3K9Ac, H3K14Ac, H3K23Ac.

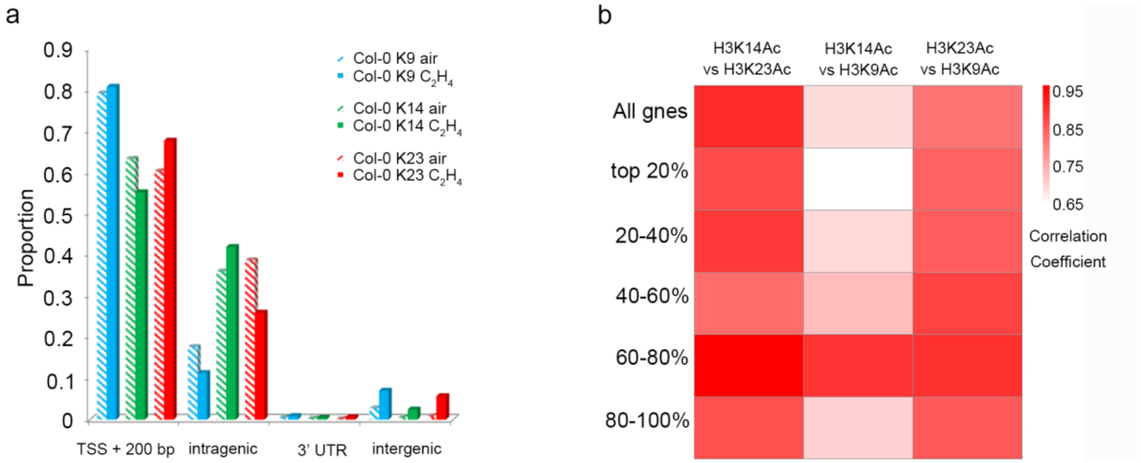

Additional file 1: Figure S3. Histone acetylation of H3K9Ac, H3K14Ac and H3K23Ac in response to ethylene. (a) Peak distributions of H3K9Ac, H3K14Ac and H3K23Ac in ethylene regulated genes at at the TSS and 200 bp downstream of the TSS (TSS+200bp), in intragenic regions (inside exons or introns excluding the peaks that overlapped with the TSS+200bp and 3' UTR), in 3' UTRs, and in intergenic regions (upstream and downstream of genes). (b) The correlation of histone enrichment (RPKM) of H3K14Ac vs. H3K23Ac; H3K14Ac vs. H3K9Ac; and H3K23Ac vs. H3K9Ac in gene body regions with transcript abundance in the presence of ethylene. "All genes" indicates the total genes in *Arabidopsis*. Genes were ranked according to relative mRNA expression levels in the presence of ethylene and divided into five equal sets according to Fig1 (d).

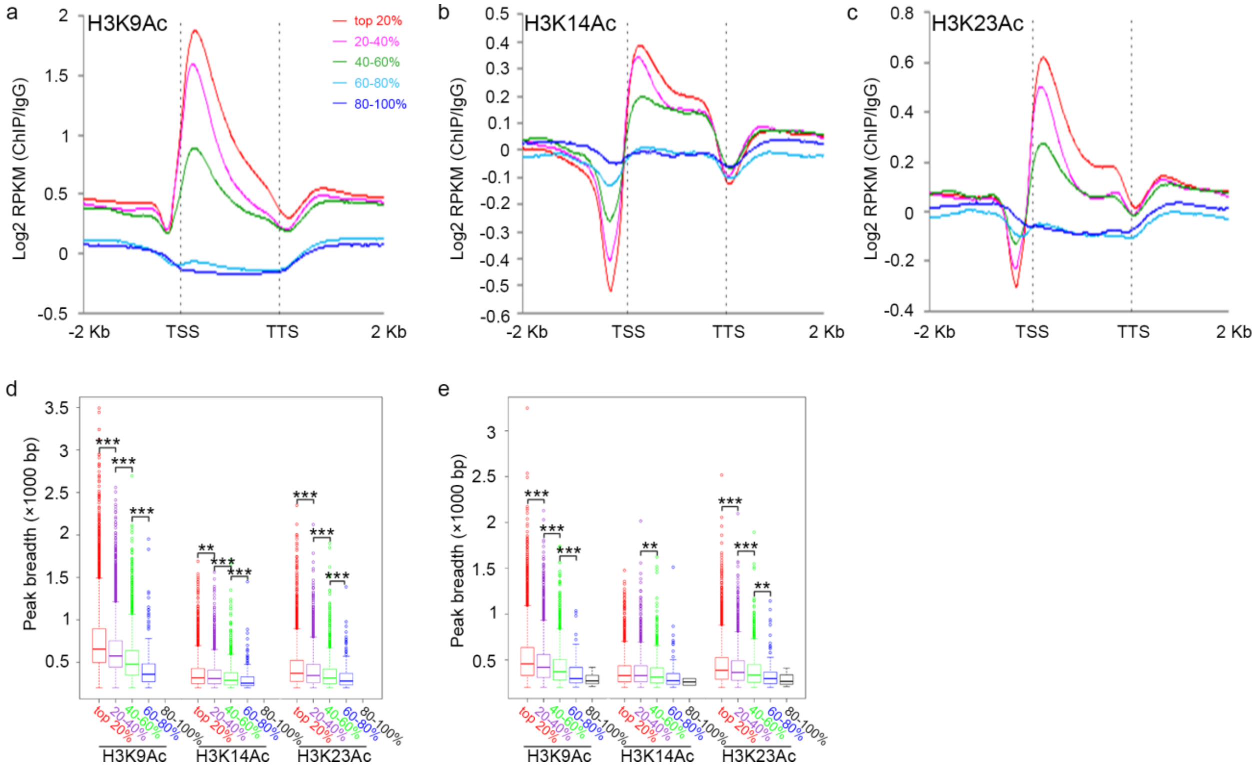

Additional file 1: Figure S4. The ethylene induced shift of histone acetylation marks is EIN2 dependent. (a-c) Mean enrichment profiles presenting the genomic distribution of reads for H3K9Ac (a), H3K14Ac (b), and H3K23Ac (c) in *ein2-5* along gene bodies. Genes were ranked according to relative mRNA expression levels and divided into five equal sets (as Fig1d). (d-e), Boxplot showing the peak sizes in air (d) and ethylene (e) condition at their associated genes. Peak associated genes were divided into five sets through overlapping with gene sets in Fig1 (d). The ** indicates P < 0.01 by t-test; the ** indicates P < 0.001 by t-test.

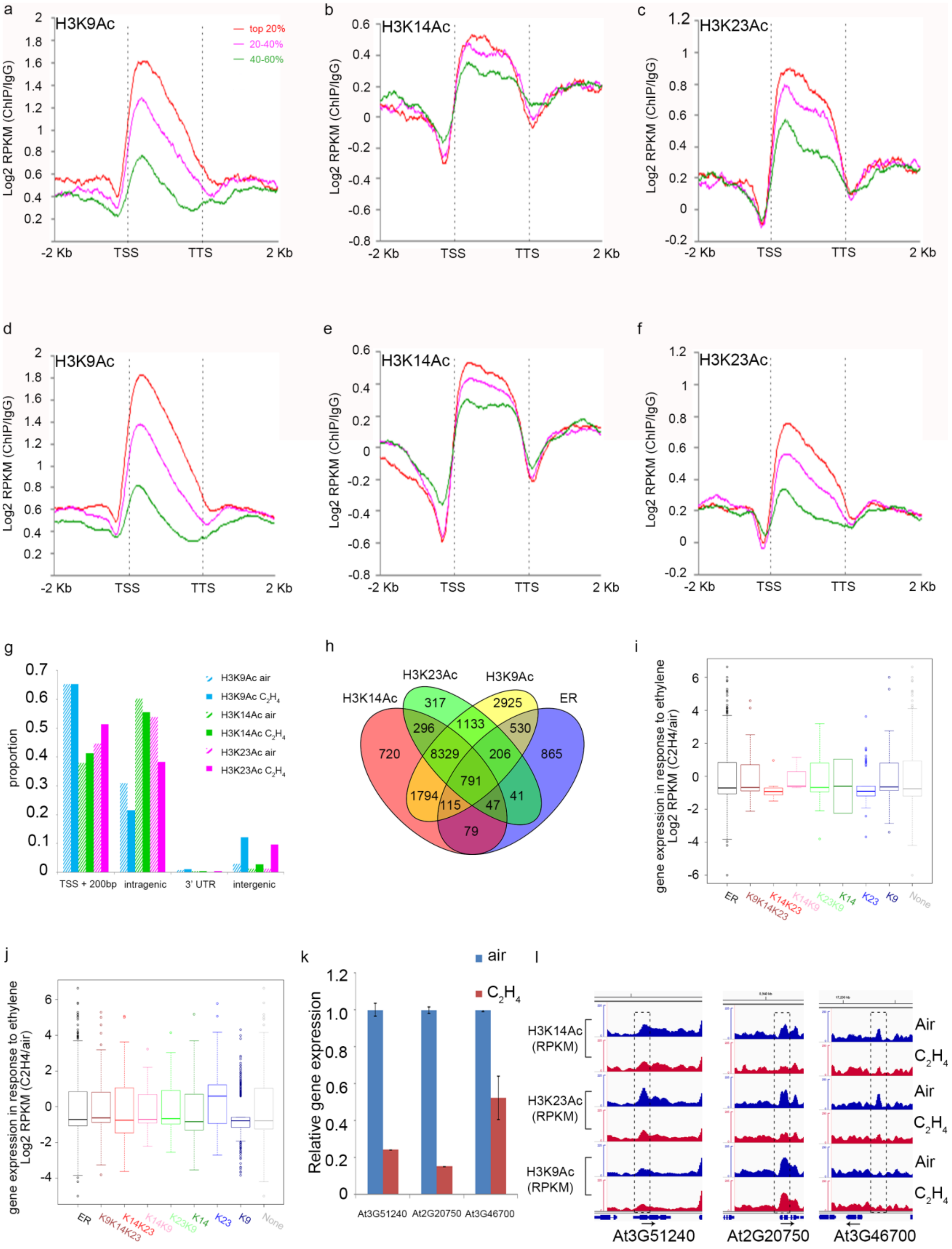
a
H3K9Ac
top 20%
20-40%
40-60%
Log2 RPKM (ChIP/IgG)
-2 Kb
TSS
TTS
2 Kb
b
H3K14Ac
c
H3K23Ac
d
H3K9Ac
e
H3K14Ac
f
H3K23Ac
g
proportion
H3K9Ac air
H3K9Ac C_2H_4
H3K14Ac air
H3K14Ac C_2H_4
H3K23Ac air
H3K23Ac C_2H_4
TSS + 200bp
intragenic
3' UTR
intergenic
h
H3K14Ac
H3K23Ac
H3K9Ac
ER
317
2925
296
1133
530
720
8329
206
865
791
1794
41
115
47
79
i
gene expression in response to ethylene
Log2 RPKM (C2H4/air)
ER
K9K14K23
K14K23
K14K9
K23K9
K14
K23
K9
None
j
gene expression in response to ethylene
Log2 RPKM (C2H4/air)
ER
K9K14K23
K14K23
K14K9
K23K9
K14
K23
K9
None
k
air
C_2H_4
Relative gene expression
At3G51240
At2G20750
At3G46700
l
H3K14Ac
(RPKM)
H3K23Ac
(RPKM)
H3K9Ac
(RPKM)
Air
C_2H_4
At3G51240
At2G20750
At3G46700

Additional file 1: Figure S5. Histone modification in ethylene regulated genes. (a-f), Mean enrichment profiles presenting the genomic distribution of histone enrichment for H3K9Ac (a, d), H3K14Ac (b, e) and H3K23Ac (c, f) along gene body without (a-c) and with ethylene (d-f) in ethylene-regulated genes. All genes in *Arabidopsis* were ranked according to relative mRNA expression levels and were divided into five equal sets, and then the ethylene-regulated genes were selected for the analysis. (g) H3K9Ac, H3K14Ac and H3K23Ac peak distribution in ethylene regulated genes at the TSS and 200 bp downstream of the TSS (TSS+200bp), intragenic, inside exons or introns excluding the peaks that overlapped with the TSS+200bp and 3' UTR; 3' UTR, and intergenic region (upstream and downstream of genes). (h) Venn diagram showing the overlap of ethylene regulated genes with H3K9Ac, H3K14Ac or H3K23Ac labeled genes in Col-0 with ethylene treatment. (i-j) Boxplot shows the correlation of changes in gene expression and histone acetylation without (i) or with the presence of ethylene (j). “ER” indicates all the differentially expressed genes in response to ethylene, n=2674; K9K14K23, peak associated genes shared among H3K9Ac, H3K14Ac and H3K23Ac, n=202 for (i), n=791 for (j); K14K23, peak associated genes specifically labeled by H3K14Ac and H3K23Ac, but not H3K9Ac, n=13 for (i), n=47 for (j); K14K9, peak associated genes specifically labeled by H3K14ac and H3K9Ac, but not H3K23Ac, n=3 for (i), n=115 for (j); K23K9, peak associated genes specifically labeled by H3K23Ac and H3K9Ac, but not H3K14Ac, n=195 for (i), n=206 for (j); K14, K23 and K9, peak associated genes uniquely labeled by K14, K23 and K9, separately,K14 n=2 for (i), n = 79 for (j), K23 n=67 for (i), n=41 for (j), K9 n=557 for (i), n=530 for (j); none, genes that could not be assigned to any peaks of H3K9Ac, H3K14Ac, H3K23Ac. (k) Quantitative RT-PCR showing the down regulation of candidate genes, which show similar pattern with the expression dynamics in RNA-seq. Col-0 seedlings grown in the dark for 3 days with or without 4 hours ethylene gas treatments were used. (l) Standard ChIP-seq assays showing that the decreased enrichment of H3K14Ac and H3K23Ac in the candidate genes (3 representative genes indicated in the figure, which validated by qPCR in (k) in Col-0 treated with ethylene and no significant enrichment of H3K9Ac is observed. Binding levels are indicated by reads per kilobase per million reads in sample (RPKM). Col-0 seedlings grown in the dark for 3 days with or without 4 h ethylene gas treatment for ChIP-seq. Arrow indicates the direction of gene. Dash boxes highlight the different enrichments of H3K9Ac, H3K14Ac or H3K23Ac.

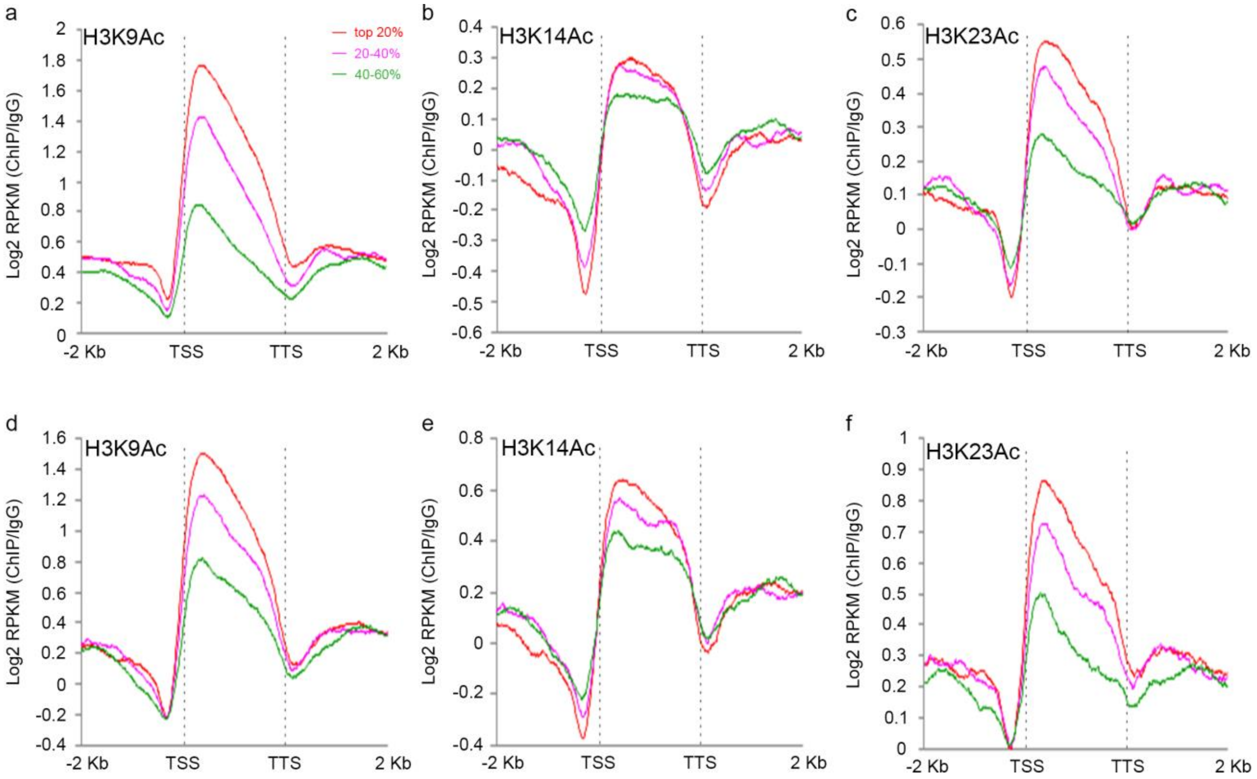

Additional file 1: Figure S6. Histone modification in ethylene regulated genes in *ein2-5*. (a-f) Mean enrichment profiles presenting the genomic distribution of reads for H3K9Ac (a, d), H3K14Ac (b, e) and H3K23Ac (c, f) in *ein2-5* without ethylene (a-c) or ethylene (d-f) treatment, which along gene body of ethylene regulated genes. Genes were ranked according to relative mRNA expression levels and divided into five equal sets, and then the ethylene regulated genes were selected for the analysis. The results from bottom two gene sets were not shown due to none or very few genes.
